# Severe Acquired Toxoplasmosis Caused by Wild Cycle of *Toxoplasma gondii*, French Guiana

**DOI:** 10.3201/eid1504.081306

**Published:** 2009-04

**Authors:** Bernard Carme, Magalie Demar, Daniel Ajzenberg, Marie Laure Dardé

**Affiliations:** Université des Antilles et de la Guyane, Cayenne, French Guiana (B. Carme); Centre Hospitalier de Cayenne, Cayenne (B. Carme, M. Demar); Faculté de Médecine de Limoges, Limoges, France (D. Ajzenberg, M.L. Dardé)

**Keywords:** Toxoplasma gondii, severe toxoplasmosis, atypical strains, epidemiology, wild cycle French Guiana, Amazonia, dispatch

## Abstract

From 1998 through 2006, 44 cases of severe primary toxoplasmosis were observed in French Guiana in immunocompetent adults. *Toxoplasma* *gondii* isolates exhibited an atypical multilocus genotype. Severe disease in humans may result from poor host adaptation to neotropical zoonotic strains of *T. gondii* circulating in a forest-based cycle.

French Guiana is a French territory in South America; the Amazon rain forest covers 92% of the territory. Toxoplasmosis is a cosmopolitan parasitic disease. It is usually benign in patients without immunosuppression, but in French Guiana, it is a major public health problem, mainly because of the high prevalence of AIDS and because of an emerging severe form of acquired toxoplasmosis in immunocompetent patients.

## The Cases

Until 1998, only 2 cases of severe primary toxoplasmosis had been reported in French Guiana, in 1992 (*1*) and 1997 (*2*). However, from 1998 through 2006, 44 cases occurred (*3*–*5*). All patients were immunocompetent (not HIV-infected) adults who had been hospitalized because of a marked, nonspecific, infectious syndrome. All patients had elevated and prolonged fever; most had weight loss, hepatic cytolysis, lymphadenopathy, headache, and pneumopathy. Other signs such as rash, retinochoroiditis, myocarditis, myositis, and neurologic disorders, may occur with toxoplasmosis, albeit infrequently. Prognosis is linked to lung involvement, which typically occurs 10–15 days after onset of fever and requires hospitalization. Approximately one third of patients with this severe form of disease experience respiratory distress and need to be in an intensive care unit. Unless specific treatment (sulfadiazine and pyrimethamine) is initiated quickly, death may occur.

In these patients, acute toxoplasmosis was diagnosed by serologic tests, which suggested recent primary infection (substantial and rapid increase in immunoglobulin [Ig] G, associated with specific IgM, in 2 separate samples taken 1–2 weeks apart tested at the same time); by blood or bronchoalveolar lavage samples positive by PCR; and by absence of an alternative cause. *Toxoplasma* *gondii* strains, virulent in mice, were isolated in some cases. Microsatellite analysis performed on isolated strains or on toxoplasma DNA extracts showed that all isolates exhibited an atypical multilocus genotype, in contrast with strains usually described in Europe or North America. Most patients reported forest-related activities such as ingestion of surface water, consumption of undercooked game meat, and hunting. Of the 44 patients, 1 died; the others recovered after standard treatment.

We recently described an outbreak of toxoplasmosis from late December 2003 through mid January 2004 involving 11 cases among the 38 inhabitants of a village in Suriname near the French Guiana border (*6*). Using 8 microsatellite markers with a high power of discrimination, we described a unique multilocus genotype for 5 patients and demonstrated that only 1 strain was responsible for this outbreak (in at least 5 of 11 patients). However, the same strain was responsible for different clinical outcomes in each of the 11 patients: 2 cases were congenital and lethal, 9 cases occurred in immunocompetent adults (5 patients, 1 of whom died, had disseminated toxoplasmosis and needed hospitalization; and 4 had less severe disease with no life-threatening signs or need for hospitalization). Genetic susceptibility of the host to this unusually severe form of toxoplasmosis may explain in part the severity of symptoms, although we observed this disease in the different ethnic groups of French Guiana (Caucasians from inland France, Creoles, Amerindians, Bushinengé, and Brazilians).

## Conclusions

Such a severe outcome in humans may be explained by poor host adaptation to emerging and highly virulent strains of *T. gondii* circulating in a forest-based cycle involving wild felids (definitive hosts) and their prey (intermediate hosts) (Figure). The high seroprevalence for noncarnivorous wild mammals in French Guiana, especially terrestrial animals such as deer, armadillos, pacas, and peccaries (*7*), suggests oral exposure to oocysts eliminated by wild felids in the absence of domestic cats (*8*). Wild felids are still numerous in French Guiana. Isolation of 1 genetically atypical *Toxoplasma* strain in a free-living jaguar (*Panthera onca*) is a further argument for the existence of a *T. gondii* wildlife cycle (*9*). Sources of contamination are uncooked meat from hunted game and river water containing oocysts excreted by wild cats.

**Figure:**
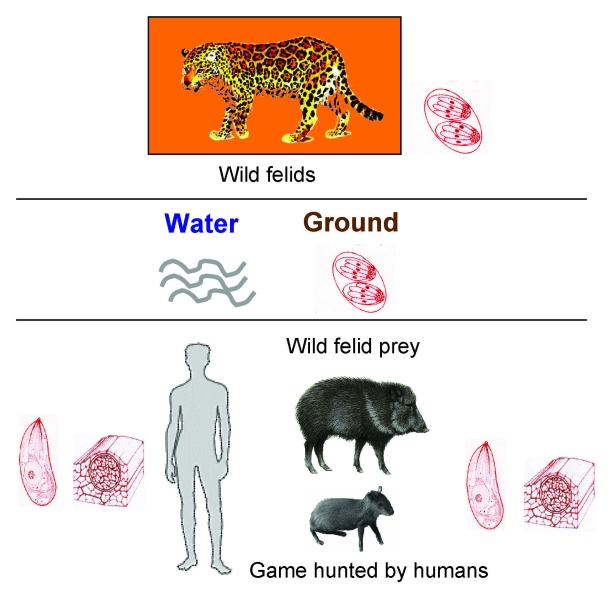
Transmission cycle of highly virulent strains of *Toxoplasma gondii* involving wild felids (definitive hosts) and their prey (intermediate hosts).

In North America and in Western Europe, *T. gondii* has been considered to have a clonal population structure and low genetic diversity; >95% of strains belonged to 3 clonal lineages, types I, II (mainly), and III (*10*). The greater genetic diversity of *T. gondii* in a wild and tropical environment, compared with the domestic environment, could be connected to the considerably higher diversity of hosts in the wild. In the neotropical rain forest, fauna are abundant and highly diverse; 560 species of mammals have been recorded (*11*). A plurality of alleles is needed for *T. gondii* to colonize a maximum of ecological niches in such a diverse environment.

Because little molecular assessment has been performed for *T. gondii* from asymptomatic infected persons in French Guiana, it is difficult to know what proportion of persons infected with the atypical strains found there have severe disease. In French Guiana, a wild cycle of *T. gondii* coexists with a domestic cycle, in which cats take a central role. Both cycles, wild and domestic, can even merge with the anthropization of the Amazonian forest. For the domestic cycle, there are 11 DNA samples (6 urban isolates from animals raised in captivity [3 cats and 3 monkeys] and 5 human isolates from patients with classical disease [2 AIDS patients with cerebral toxoplasmosis, 2 patients with congenital infections, and 1 patient with lymphadenopathy]). These samples have less allelic diversity than wild isolates.

Recent studies that used multiple markers and collected samples from other countries have shown that, at least in the tropical part of South America, *T. gondii* strains have higher genetic diversity (*12*,*13*). Few data concerning the genetic diversity and population structure of this parasite are available in Africa (*14*) and Asia (*15*). The number of cases of this severe form of primary *T. gondii* infection (>50 well-documented observations in the past decade) is particularly high in French Guiana, given the small size of the population (≈200,000 inhabitants), compared with the low number of published cases.

Although such a wild *T. gondii* cycle with severe human clinical consequences has not been described in Brazil or in other countries of the Guyana Shield, it seems unlikely that it is a disease specific to French Guiana. Amazonian areas, and likely other rain forest areas where felids live, could be affected by this form of toxoplasmosis, which could be designated “Amazonian toxoplasmosis” or “wild rain forest toxoplasmosis.”

From a healthcare perspective worldwide, physicians should consider a diagnosis of acute toxoplasmosis as soon as possible after the onset of signs and symptoms in patients who live in or have recently visited the Amazonian region and who have a severe infectious syndrome with visceral, especially lung, involvement. Serologic tests should be promptly submitted for such patients. If recent infection with *T. gondii* is diagnosed, a potent antitoxoplasmosis treatment (sulfadiazine plus pyrimethamine) must be prescribed without delay.
